# Heatwave 1987: the Piraeus *versus* Athens case

**DOI:** 10.12688/f1000research.124999.1

**Published:** 2023-01-31

**Authors:** Stella Geronikolou, Stelios Zimeras, Stephanos Tsitomeneas, George P Chrousos

**Affiliations:** 1 Clinical, Translational and Experimental Surgery Research Centre, Biomedical Research Foundation Academy of Athens, Athens, 11527, Greece; 2 University Research Institute of Maternal and Child Health & Precision Medicine, National and Kapodistrian University of Athens, Athens, 11527, Greece; 3 UNESCO Chair of Adolescent Health, National and Kapodistrian University of Athens, Athens, 11527, Greece; 4 Mathematics & Statistics, University of Aegean, Samos, Greece; 5 University of West Attica, Athens, Greece

**Keywords:** heatwaves; Eastern Mediterranean Sea; mortality; heatwave 1987; Athens 1988; Athens 1992; odds ratio; relative risk; mortality; neural networks; ozone; NO

## Abstract

**Background:** Heatwaves represent the main indices of climate change, while mortality is one of the established markers of their human effects. Populations adapt to temperature variations/challenges differently for unknown reasons. Thus, heatwave evaluations should be enriched by historical context and local data, to allow better precision and prediction.

**Methods:** The mortality data for 1987 were collected from the Piraeus municipality registry, whereas data for Athens were obtained from literature retrieved from PUBMED. Ambient characteristics were derived from Geronikolou’s 1991 thesis and the reports of national organizations. From the death events, the odds ratio and relative risk in Athens compared to the Piraeus 1987 event were calculated. Finally, a simple neural network proposed the dominant ambient parameter of the heatwave phenomenon in each place.

**Results:** The 1987 heatwave was more lethal (seven-fold) in Athens than in Piraeus and was found to be dependent on nitric oxide (NO) concentration (with probability 0.999). In the case of Piraeus in 1987, ozone characterized the phenomenon.

**Conclusions:** The odds of dying due to a heatwave are highly dependent on lifestyle, population sensitivity to preventive measures and public health policy, while the phenomenon was mainly moderated by ozone in Piraeus in 1987, and NO in Athens irrespective of year.

## Introduction

Excessive ambient temperatures represent the main indices of climate change, while mortality is one of the most established markers of its human effects. In the last few decades, the annual incidence of extreme weather event disasters has tended to rise ^1^. Thus, in the last 50 years, 22,173 all-cause disasters (conflicts, biological, natural, technological) have taken place, causing 6.2 million deaths globally ^2^. The same report also suggests that 8% of the 89% of the observed total all-cause mortalities that were registered in the Emergency Events Database (EM-DAT) of the Center for Research on the Epidemiology of Disasters (CRED) ^1, 2^ were attributed to heatwaves ^1^.

High ambient temperatures are closely related to mortality increases in most countries ^3^, and this risk is expected to rise in the near future, as they are closely related to climate change ^4– 7^. Daily deaths and high ambient summer temperatures have been correlated irrespective of city heterogeneity, as shown in the Assessment and Prevention of Acute Health Effects and Weather Conditions in Europe (PHEWE) project ^8, 9^. Daily deaths and weather and air quality data for 15 European cities were analyzed over 11 years, and it was concluded that the warm season heat-mortality curve was J-shaped, with Mediterranean cities having higher temperature thresholds and steeper slopes than Central and Northern European cities.

Populations adapt to temperature variations or challenges differently, for unknown reasons ^10^. Location, lifestyle, genetic predisposition, environmental pollution are ongoing research targets in our attempts to calculate their impact on individuals and populations. Such data are important for authorities to make correct decisions regarding public health and city planning. Public health measures should adapt to these considerations in order to appropriately mitigate heatwave effects. The evaluation of heatwaves should be enriched by historical context and local events to allow better precision in data analysis and more accurate predictions of future crises. This study aims to contribute with a heatwave report for Piraeus in 1987 published as part of a dissertation in the Public Hygiene Department ^11^, as this is a representative urban population (ranked third in growth) and the biggest port in the Mediterrannean Sea. The Piraeus 1987 case will be examined it in the light of the mortality odds, and more importantly, will be compared with other heatwaves in the same geographical area at the same and different times.

## Methods

A retrospective study was designed comprising of the daily deaths of all causes during the summer of 1987 in Piraeus ^11^. The above heatwave was compared to diverse heatwaves in the Attica area: Athens in 1987 ^12– 14^, Athens in 1988 ^12– 14^, and Athens in 1992 ^15^.

The data extracted by these studies were processed to calculate:

•   the odds ratio as follows: OR=π/(1-π), where π: incidence (risk) ^16^.

•   the relative risk of dying in each heatwave (v) compared to the Piraeus 1987 heat wave mortality risk:

RR= π _V_/π _Piraeus _^16^

Discomfort index (DI) is an index of the discomfort felt in warm weather as a result of the combined effects of the temperature and air humidity. It was calculated using Thom’s formula ^17^ as follows:

DI=T- 54*(0.55-0.0055*RH)*(T-14.5),

where T: temperature; RH: relative humidity.

Ozon and nitric oxide (NO) measurements derived either from the included studies and/or 

We also collected and present pollution and solar activity data (sunspot numbers) for each month that the heatwave under investigation had been observed. The sunspot numbers are related to cosmic rays and are investigated for their influence on the climate and for their impact on health ^18^.

## Results

Daily mortality events were based on Geronikolou’s (1991) thesis that included a study of all death events archived in the city of Piraeus between June 1 ^st^ 1987 and August 31 ^st^ 1987. In these 92 days, 263 death events were archived, with 62 of them occurring during the heatwave ^11^. Concerning the Athens data, they were extracted by the publications ^12– 15^.

In Table 1 the registered population in each municipality census (for both Piraeus and Athens) are listed

**Table 1.** Cities of Piraeus and Athens demographics.

Year	Piraeus	Piraeus greater area	Athens
**1951**	186,088	n/a	n/a
**1961**	183,957	n/a	n/a
**1971**	187,458	439,138	867,023
**1981**	196,389	476,304	885,737
**1991**	182,671	456,865	772,072
**2001**	175,697	466,065	745,514
**2011**	163,688	448,997	664,046

n/a: not available; Source: Hellenic National Statistic Authority

In Table 2 the geography and pollution characteristics and the calculated DI are illustrated.

**Table 2.** Ambient characteristics in Piraeus and Athens due to heatwaves.

Characteristics	Piraeus 1987	Athens 1987	Athens 1988	Athens 1992
**Latitude**	37.873°	37.984	37.984	37.984
**Longitude**	23.675°	23.7275	23.7275	23.7275
**NO (μg/m ^3^)**	88	162	182	n/a (157)
**Ozon (μg/m ^3^)**	93	87	86	84
**Temperature (OC)**	42	43.6	42	41.6
**Humidity (%)**	57.33	60	n/a	n/a
**Discomfort index**	37.077	37.2	n/a (37.33)	n/a (37.46)
**Duration (days)**	10	10	7	n/a
**Sunspot numbers**	33	33	39	64.4

n/a: not available

In Table 2, to overcome the problem with the missing values (noted as n/a), two interpolation methods were calculated considering:

1. Lagrange interpolation polynomial ^19– 21^ and

2. Cubic spline interpolation ^21, 22^.

Due to the uncertainty in the form (direction) of the data, the proposed method is the computation of the average between the two interpolation methods. Results are as illustrated inside the bracket of the data.

In Table 3 the calculated probabilities of heat-related mortality are presented.

**Table 3.** Calculated probabilities of mortality- risk in Piraeus and Athens due to heatwaves.

Mortality/heatwave	Piraeus 1987	Athens 1987	Athens 1988	Athens 1992
**Deaths (absolute)**	67	2000	28	359
**Odds Ratio (OR) ^#^**	0.0003157	0.002258007	0.00000316121	0.000464983
**Relative risk (RR) ^#^**	1	7.152382	0.100133	1.472862

# : Calculations are based on dead/survived+dead

Usually, neural networks are applied to big data analysis cases, but in this case, their use is as a pilot for data processing. Regarding the effect of the factors influencing the occurrence of heatwaves, a simple neural network- developed taking into consideration the proposing parameters from Table 2: NO, ozone, temperature, discomfort index and sunspot. The idea behind this model is to predict the importance of the parameters affecting the heatwaves. The model x _i _i=1,..,n consists of the proposing parameters and w _i _i=1,..,n represents the corresponding weights. In our case all the parameters have the same weight (w _i _=1);

Σ is denoted as the summation of the multiplication between parameters and weights (w _i _x _i_); f(x) is the activation function.

The activation function is an integral part of a neural network. Without an activation function, a neural network is a simple linear regression model. This means the activation function gives non-linearity to the neural network. The proposed formula for the function is the softmax with [33–36]


$$Soft\;\max(x_{i})=\frac{e^{x_{i}}}{\sum_{i=1}^{k}e^{x_{i}}},\;j=1,2,\ldots ,k$$

The exponential acts as the non-linear function. The values were divided by the sum of exponential values to normalize and then convert them into probabilities.

**Figure 1.**
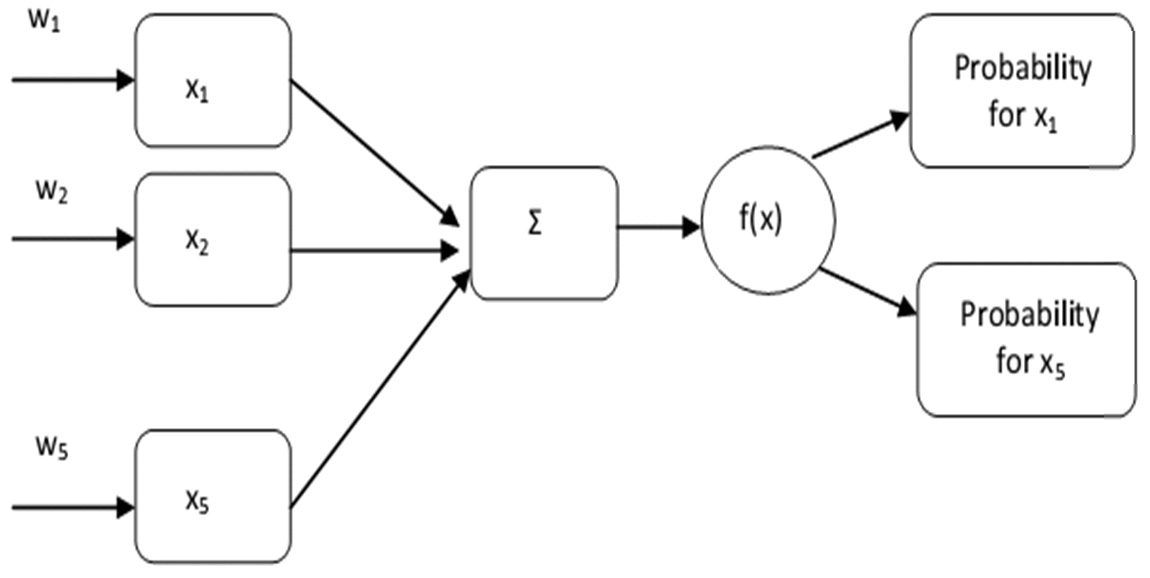
Neural network.

Based on the proposed neural network ( Figure 1) the important parameters for different years are:

Piraeus 1987 - ozone probability 0.993

Athens 1987 - NO probability 0.999

Athens 1988 - NO probability 0.999

Athens 1992 - NO probability 0.999

## Discussion

**Figure 2.**
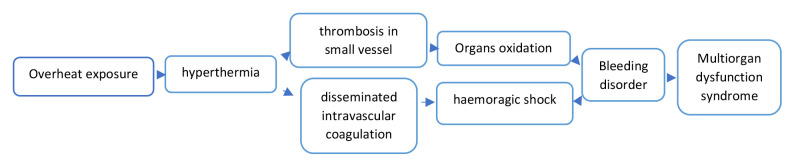
Physiologic mechanisms explaining heat-related morbidity and mortality.

The Eastern Mediterranean Sea is surrounded by highly diverse regions as North Africa, the Middle East and Asia Minor. Socioeconomic, environmental and population inequities have been associated with anthropogenic dryness and climate change. For millennia, ambient temperature exposure influences the human body leading to physiologic responses that may be even morbid or lethal ( Figure 2). The ancient Greek physician Hippocrates, known as the father of Western medicine, in his work *“On airs, waters and places”*, documented the human physiology and clinical manifestaions variations amid populations living under different environmental conditions (wind flow, density and direction, soil type, temperature, humidity, waters, etc.). On the other hand, Roman mythology, by attributing the heat phenomena to the rise of the “dog star” (Sirius) defined the time periods of heatwaves between 3 ^rd^ of July and 11 ^th^ of August each year ^12^. Coincidentally, the heat events studied herein took place during July. Currently, we have real-time and historical records to evaluate the heatwave effects and plan future mitigations via public health measures.

In this study, we focus on the largest port of Southeast Europe and the Eastern Mediterranean Sea, Piraeus. According to the National Oceanic and Atmospheric Administration (NOAA), the location’s latitude is 37.873° and longitude is 23.675, while its coordinates are 37°56′34.8″N 23°38′49″E. It is a rather flat (highest elevation/hill) 87 m (285 ft) coastal urban city covering 50.417 km ^2^ (19.466 sq mi). Piraeus’s seaport is only 7 km from the Athens city center. It usually has temperature which is 3 °C lower than Athens, with blowing sea winds and a higher humidity ^12, 13^. Piraeus is flat, including only one hill of 87 m height, while the mountains are in the far distance. Athens, on the contrary, includes hills and mountains, and is characterized by vast urban density and numerous heat-islands due to stone pedestrian areas, narrow roads and high buildings. In Piraeus the urban density is clearly lower and the roads are wider, allowing the sea winds to cool the city. In addition, the sea water vapor, enhanced by extreme heat, contributes to the deleterious effects of carbon dioxide while being transported inland by the blowing western winds ^23^ It should be noted herein that this water vapor percipitates by 0.04 p/million annually ^23^.

The seven-fold relative risk in mortality of Athens compared to Piraeus in 1987 may be explained by the geography (Athens city basin is surrounded by four mountains and includes four hills), the urban density, and the microclimatic characteristics (sea jets, mostly plain ground, etc). The photochemistry pollution emitted by the industries located in the greater Piraeus area (beyond the Piraeus municipality borders) is transported to Athens city center by the western wind and/or jets, possibly influencing the population, but not the surface ozon concentration, as established by Varotsos *et al.* ^24^ The same study and Kiraly ^25^ suggested that temperature, wind flows and humidity may be responsible for the *in situ* variations of pollution and relevant episodes. Although the calculated discomfort index was at medical emergency levels (> 32) and more or less equivalent in Piraeus and Athens, the deaths observed were clearly fewer than those that occurred in the Athens municipality ( Table 2). 

The differences in death events between the Athenian heatwaves are attributed to preventive measures and daily practice followed by the population. Most, if not all, houses took sun shading and air conditioning provisions immediately following the 1987 heat. Greek authorities imposed restrictions on outdoor working hours (for blue collar workers, construction workers, farmers, etc.) and advised the population to stay at home or under shade during the most dangerous hours of 11am–4pm.

Pollution parameters are directly and indirectly affected by solar activity ^26^. Solar activity contributes to stroke-related mortality (thrombotic events), as demonstrated by previous studies ^27^, although it has been established that it does not to climate change ^26^. Thus, we added the relevant data, herein, for future consideration with future heatwave events. Finally, the 1987 heatwave results were of great importance because confounding factors, such as lifestyle, dietary preference variations, high pollution exposure, and genetic heterogeneity were absent at the time ^27^. Our study was limited to death events reported by the municipality and not the greater area of Piraeus or Athens. The health impacts of exposure to overheating include hyperthermia, triggering bleeding disorder either through thrombosis in small vessels or disseminated intra-vascular coagulation. The resultant bleeding disorder may lead to multi-organ dysfunction syndrome and/or death ( Figure 2).

The 1987 heatwave in Greece had been a real milestone in public health policy and citizens’ daily practice. Until this heatwave, construction practices followed a certain manner, which neglected traditional methods which were used to protect from prolonged extreme weather events in the past. More specifically, citizens had no provision for sun shading or air conditioning. After the 1987 heatwave, the Greek authorities and the population took measures to better prepare against such events in the future. This was mirrored in the lower mortality rates due to extreme heat events in 1988 and 1992. Some of the excess mortality risk (about 47%) observed in 1992 is probably explained by a significant change in population consistency as a result of a vast immigration flow from the northern borders of the country, that was in progress at the time and not registered in the 1991 census.

Finally, these results confirm the findings of Dimitriadou *et al.,* Ebi *et al.* and Mazaraki *et al.,* indicating that public health warning systems need to be created which consider locality, including geography, microclimatic parameters, population consistency and behavior ^28– 30^.

The probability of NO affecting each heatwave was found to be higher in Athens irrespective of year. The lack of significant fluctuations of NO in time has been noted by Varotsos ^24^. In Piraeus 1987, ozone probability (certainty) to regulate the heatwave effect on strokes was higher. Additionally, it is established that ozone levels show a positive linear correlation to ambient temperature ^31^, man-made pollution and moisture ^32^. Thus, we believe that, during the Piraeus heatwave in 1987, seawater evaporated, entraining industrial pollution and subsequently increasing tropospheric ozone through photochemical reactions. 

## Conclusions

The 1987 heatwave had a decisive role in Greek reality. From the same heat-stress event, Piraeus, a coastal city, experienced less mortality than the neighboring continental Athens. The heatwave events that followed had milder effects because of the newly established public health prevention policy and citizens’ adaptation to the new measures. The heatwaves were affected by NO in Athens every year and ozone in Piraeus in 1987, as observed using probabilities extracted via network evaluations.

## Data Availability

Piraeus Mortality data may be found in Geronikoulou *et al.* ^27^. The rest of the mortality data is derived from the included publications. All pollution measurements (ozone and nitric oxide) were calculated from the measurements included in the Hellenic Ministry of Environment and Energy reports, whereas humidity and temperature measurements were derived from literature as well as the Hellenic National Meteorological Service archives Figure 1 Our created network Figure 2 Our created summary of heat-related physiology mechanisms for non-health educated readers convenience
