# Supplementary material for: Heatwave 1987: the Piraeus versus Athens case
Source: F1000Res. 2024 Feb 29;12:115. Originally published 2023 Jan 31. [Version 2] doi: 10.12688/f1000research.124999.2 (PMC10907872; doi:10.12688/f1000research.124999.2)
Supplement: Peer review history

# Reviewer report: Reviewer response for version 2

**DOI:** 10.5256/f1000research.157393.r251741

**Published:** 2024-03-01

**Authors:** Themis P. Exarchos

**Affiliations:** 1 Bioinformatics and Human Electrophysiology Laboratory, Department of Informatics, Ionon University, Corfu, Greece

Authors have addressed all my concerns.

Is the work clearly and accurately presented and does it cite the current literature?

Yes

If applicable, is the statistical analysis and its interpretation appropriate?

Yes

Are all the source data underlying the results available to ensure full reproducibility?

Yes

Is the study design appropriate and is the work technically sound?

Yes

Are the conclusions drawn adequately supported by the results?

Yes

Are sufficient details of methods and analysis provided to allow replication by others?

Yes

Reviewer Expertise:

Data mining, decision support systems

I confirm that I have read this submission and believe that I have an appropriate level of expertise to confirm that it is of an acceptable scientific standard.

# Reviewer report: Reviewer response for version 1

**DOI:** 10.5256/f1000research.137258.r161963

**Published:** 2023-09-08

**Authors:** Themis P. Exarchos

**Affiliations:** 1 Bioinformatics and Human Electrophysiology Laboratory, Department of Informatics, Ionon University, Corfu, Greece

This article presents an analysis and comparison of the heatwaves of Piraeus and Athens in 1987. Mortality data were collected from registries and literature, whereas ambient characteristics were collected by previous research and open national reports.

 The odds ratio of death events of the two cities were calculated and compared.

 The major outcome found was that the heatwave was seven-fold more lethal in Athens than in Piraeus, dependent on nitric oxide (NO) concentration.

 The article is well written and results are presented in an easy to understand way for the readers.

Comments to the authors:

 Please extend the introduction section with additional references and state of the arts in similar works (other heatwaves, other parameters taken into account, comparison models etc).

 In addition, please add some more details on the identification of the following probabilities:

 Piraeus 1987 - ozone probability 0.993

 Athens 1987 - NO probability 0.999

 Athens 1988 - NO probability 0.999

 Athens 1992 - NO probability 0.999

Is the work clearly and accurately presented and does it cite the current literature?

Yes

If applicable, is the statistical analysis and its interpretation appropriate?

Yes

Are all the source data underlying the results available to ensure full reproducibility?

Yes

Is the study design appropriate and is the work technically sound?

Yes

Are the conclusions drawn adequately supported by the results?

Yes

Are sufficient details of methods and analysis provided to allow replication by others?

Yes

Reviewer Expertise:

Data mining, decision support systems

I confirm that I have read this submission and believe that I have an appropriate level of expertise to confirm that it is of an acceptable scientific standard, however I have significant reservations, as outlined above.

# Response

**Published:** 2023-10-25

**Authors:** Stella Geronikolou

This article presents an analysis and comparison of the heatwaves of Piraeus and Athens in 1987. Mortality data were collected from registries and literature, whereas ambient characteristics were collected by previous research and open national reports.

 The odds ratio of death events of the two cities were calculated and compared.

 The major outcome found was that the heatwave was seven-fold more lethal in Athens than in Piraeus, dependent on nitric oxide (NO) concentration.

 The article is well written and results are presented in an easy to understand way for the readers.

Comments to the authors:

 Please extend the introduction section with additional references and state of the arts in similar works (other heatwaves, other parameters taken into account, comparison models etc).

*We have added 3 more references (11-13) targeting to the same nearer-geographical area, as well as one (10) that establishes the need for studies like ours.*

 In addition, please add some more details on the identification of the following probabilities:

 Piraeus 1987 - ozone probability 0.993

 Athens 1987 - NO probability 0.999

 Athens 1988 - NO probability 0.999

 Athens 1992 - NO probability 0.999

*We added the following text:*

*The values were divided by the sum of exponential values and then converted into probabilities. According to previous analyses, the opted softmax function translated the resultiant numbers into a probability distribution. Thus, the output of the function can be interpreted by a percentage number representing the probability of an event to occur.*

# Reviewer report: Reviewer response for version 1

**DOI:** 10.5256/f1000research.137258.r185613

**Published:** 2023-07-28

**Authors:** Alexandros G. Georgakilas

**Affiliations:** 1 DNA Damage Laboratory, Physics Department, School of Applied Mathematical and Physical Sciences, National Technical University of Athens (NTUA), Athens, Greece

In this study, the authors have studied the important role of heatwaves due to climate change. Heatwave associated mortality is one of the established markers of their human effects. Populations adapt to temperature variations/challenges differently for unknown reasons. Thus, heatwave evaluations should be enriched by historical context and local data, to allow better precision and prediction. The authors, present mortality data for 1987 collected from the Piraeus municipality registry, whereas data for Athens were obtained from literature retrieved from PUBMED. Ambient characteristics were derived from a thesis and the reports of national organizations. From the death events, the odds ratio and relative risk in Athens compared to the Piraeus 1987 event were calculated. Finally, a simple neural network proposed the dominant ambient parameter of the heatwave phenomenon in each place.

 The study is well-thought and organized but the authors need to consider the following :

 Comments:

1. Language in general must be more carefully looked at and read for more proper English. 
2. Please rephrase the sentence "The same study and Kiraly". 
3. If there is a detailed chart/map (from National Metereological Agency in Greece: EMY) of the winds blowing in the area during the heat wave of 1987 which is maybe not available on the internet, this reviewer thinks it's worth to provide these data since it will support the claims of the work.
4. Please explain why the sunspots were included and if there is any correlating data or literature that mention in 1987 in Athens and Piraeus areas morbidity rates for elderly people due to possible exposure to the high temperatures during not recommended hours like between 12-5.
5. The PubMed database - does it contain any relative biological data for such conditions?

Is the work clearly and accurately presented and does it cite the current literature?

Yes

If applicable, is the statistical analysis and its interpretation appropriate?

Yes

Are all the source data underlying the results available to ensure full reproducibility?

Yes

Is the study design appropriate and is the work technically sound?

Yes

Are the conclusions drawn adequately supported by the results?

Yes

Are sufficient details of methods and analysis provided to allow replication by others?

Yes

Reviewer Expertise:

Applied Physics and Biological effects of radiations from UV to ionizing.

I confirm that I have read this submission and believe that I have an appropriate level of expertise to confirm that it is of an acceptable scientific standard.

# Response

**Published:** 2023-10-25

**Authors:** Stella Geronikolou

In this study, the authors have studied the important role of heatwaves due to climate change. Heatwave associated mortality is one of the established markers of their human effects. Populations adapt to temperature variations/challenges differently for unknown reasons. Thus, heatwave evaluations should be enriched by historical context and local data, to allow better precision and prediction. The authors, present mortality data for 1987 collected from the Piraeus municipality registry, whereas data for Athens were obtained from literature retrieved from PUBMED. Ambient characteristics were derived from a thesis and the reports of national organizations. From the death events, the odds ratio and relative risk in Athens compared to the Piraeus 1987 event were calculated. Finally, a simple neural network proposed the dominant ambient parameter of the heatwave phenomenon in each place.

 The study is well-thought and organized but the authors need to consider the following:

 Comments:

1. Language in general must be more carefully looked at and read for more proper English. 

           
*We have appropriately edited the MS.*

1. Please rephrase the sentence "The same study and Kiraly". 
* We rephrased as follows: “This study same as that of Kitaly et al …”*
2. If there is a detailed chart/map (from National Metereological Agency in Greece: EMY) of the winds blowing in the area during the heat wave of 1987 which is maybe not available on the internet, this reviewer thinks it's worth to provide these data since it will support the claims of the work.

*Actually, there is such a map in the National Metereological Agency in Greece (EMY) in printed but not digital archives.  We have added it as Figure 3.tiff*


*  *

1. Please explain why the sunspots were included and if there is any correlating data or literature that mention in 1987 in Athens and Piraeus areas morbidity rates for elderly people due to possible exposure to the high temperatures during not recommended hours like between 12-5.T *he underlying mechanism linking solar cycle length and earth surface temperature is poorly understood.  Total solar irradiance as a valid marker of solar energy increases in solar activity maxima (sunspots increase) and decreases in solar minima accordingly.   Lane et al suggested that the sensitivity of the climate to the solar irradiance is 27% higher than its sensitivity to greenhouse warming effec*ts. 

   http://www-das.uwyo.edu/~geerts/cwx/notes/chap02/sunspots.html

*Lane, L.J., M.H. Nichols, and H.B. Osborn 1994: Time series analyses of global change data. Environ. Pollut., **83**, 63-68.*

*Aquila, V., Swartz, W. H., Waugh, D. W., Colarco, P. R., Pawson, S., Polvani, L. M., & Stolarski, R. S. (2016). Isolating the roles of different forcing agents in global stratospheric temperature changes using model integrations with incrementally added single forcings. Journal of Geophysical Research: Atmospheres, 121(13), 8067–8082. https://doi.org/10.1002/2015JD023841*

*Kopp, G., Krivova, N., Wu, C.J. et al. The Impact of the Revised Sunspot Record on Solar Irradiance Reconstructions. Sol Phys **291**, 2951–2965 (2016). https://doi.org/10.1007/s11207-016-0853-x*

*Geronikolou S, Leontitsis A, Petropoulos V, Davos C, Cokkinos D, Chrousos G. Cyclic stroke mortality variations follow sunspot patterns. F1000Res. 2020 Sep 3;9:1088. doi: 10.12688/f1000research.24794.2. PMID: 33224479; PMCID: PMC7667520.*

  

1.
  1. The highest temperature is observed between the hours 12.00-17.00. *No death event was observed in ages 0-50 y in Piraeus. Most deaths occurred in the age groups over 65 in Piraeus during this heatwave. Greek women  usually buy bread before lunchtime (from 12.00-15.00), thus the idea of the Reviewer seems real. *

The PubMed database - does it contain any relative biological data for such conditions?

 Many heatwaves have been reported in the PubMed database. No other publication refers to Southeastern Mediterranean cities before 1992.  

 We added a new citation, that of *Geronikolou S, Leontitsis A, Petropoulos V, Davos C, Cokkinos D, Chrousos G. Cyclic stroke mortality variations follow sunspot patterns. F1000Res. 2020 Sep 3;9:1088. doi: 10.12688/f1000research.24794.2. PMID: 33224479; PMCID: PMC7667520.*

*where we explain how electromagnetic perturbations affect human physiology: by a preserved physiology mechanism in ages and species (R1).*
